# The Patient Remote Intervention and Symptom Management System (PRISMS) – a Telehealth- mediated intervention enabling real-time monitoring of chemotherapy side-effects in patients with haematological malignancies: study protocol for a randomised controlled trial

**DOI:** 10.1186/s13063-015-0970-0

**Published:** 2015-10-19

**Authors:** Sibilah Breen, David Ritchie, Penelope Schofield, Ya-seng Hsueh, Karla Gough, Nick Santamaria, Rose Kamateros, Roma Maguire, Nora Kearney, Sanchia Aranda

**Affiliations:** Department of Cancer Experiences Research, Peter MacCallum Cancer Centre, East Melbourne, VIC Australia; Department of Nursing, University of Melbourne, Melbourne, VIC Australia; The Sir Peter MacCallum Department of Oncology, University of Melbourne, Melbourne, VIC Australia; Cancer Council Australia, New South Wales, Australia; Division of Haematology/Medical Oncology, Peter MacCallum Cancer Centre, East Melbourne, VIC Australia; Clinical Haematology & Bone Marrow Transplant Unit, The Royal Melbourne Hospital, Grattan Street, Parkville, VIC Australia; Department of Medicine, University of Melbourne, Melbourne, VIC Australia; Department of Psychology, Swinburne University of Technology, Hawthorn, VIC Australia; Centre for Health Policy, Melbourne School of Population and Global Health, University of Melbourne, Melbourne, VIC Australia; Royal Melbourne Hospital, Grattan Street, Parkville, VIC Australia; School of Health Sciences, University of Surrey, Guildford, UK

**Keywords:** Telehealth, ASyMS, Nursing, Chemotherapy, Side-effects, Cancer, Lymphoma, Leukaemia, Protocol

## Abstract

**Background:**

Outpatient chemotherapy is a core treatment for haematological malignancies; however, its toxicities frequently lead to distressing/potentially life-threatening side-effects (neutropenia/infection, nausea/vomiting, mucositis, constipation/diarrhoea, fatigue). Early detection/management of side-effects is vital to improve patient outcomes, decrease morbidity and limit lengthy/costly hospital admissions. The ability to capture patient-reported health data in real-time, is regarded as the ‘gold-standard’ to allow rapid clinical decision-making/intervention. This paper presents the protocol for a Phase 3 multi-site randomised controlled trial evaluating a novel nurse-led Telehealth intervention for remote monitoring/management of chemotherapy side-effects in Australian haematological cancer patients.

**Methods/Design:**

Two hundred and twenty-two patients will be recruited from two hospitals. Eligibility criteria include: diagnosis of chronic lymphocytic leukaemia/Hodgkin’s/non-Hodgkin’s lymphoma; aged ≥ 18 years; receiving ≥ 2 cycles chemotherapy. Patients will be randomised 1:1 to either the control or intervention arm with stratification by diagnosis, chemotherapy toxicity (high versus low), receipt of previous chemotherapy and hospital. Patients allocated to the control arm will receive ‘Usual Care’ whilst those allocated to the intervention will receive the intervention *in addition* to ‘Usual Care’. Intervention patients will be provided with a computer tablet and software prompting twice-daily completion of physical/emotional scales for up to four chemotherapy cycles. Should patient data exceed pre-determined limits an Email alert is delivered to the treatment team, prompting nurses to view patient data, and contact the patient to provide clinical intervention. In addition, six scheduled nursing interventions will be completed to educate/support patients in use of the software. Patient outcomes will be measured cyclically (midpoint and end of cycles) via pen-and-paper self-report alongside review of the patient medical record. The primary outcome is burden due to nausea, mucositis, constipation and fatigue. Secondary outcomes include: burden due to vomiting and diarrhoea; psychological distress; ability to self-manage health; level of cancer information/support needs and; utilisation of health services. Analyses will be intention-to-treat. A cost-effectiveness analysis is planned.

**Discussion:**

This trial is the first in the world to test a remote monitoring/management intervention for adult haematological cancer patients receiving chemotherapy. Future use of such interventions have the potential to improve patient outcomes/safety and decrease health care costs by enabling early detection/clinical intervention.

**Trial registration:**

ACTRN12614000516684.

Date registered: 12 March 2014 (registered retrospectively).

## Background

Haematological malignancies (including lymphomas and leukaemias) account for around 10 % of new cancer diagnoses in Australia [1] and are the sixth most prevalent form of cancer overall, with lymphomas being the most commonly diagnosed cancer in the 15–24 year age-group [1]. Chemotherapy is a core treatment for haematological malignancies; however, its toxicities often lead to potentially life-threatening or distressing side-effects (eg febrile neutropenia, infections, mucositis, nausea, vomiting, fatigue) [2–6]. Toxicities such as mucositis are more commonly experienced in haematological maligancies than solid tumours [6] and are associated with poorer treatment adherence, impaired quality of life, increased infections, mortality, hospitalisations and ultimately with a higher cost/economic burden to the health care system [7–12].

### Need for real-time assessment of side-effects

The early detection of side-effects such as severe mucositis and febrile neutropenia is vital, as they are potentially life-threatening and often lead to costly hospital admissions and higher patient morbidity, especially in haematological cancers [2, 7, 8, 12]. Management of non-life- threatening side-effects, such as fatigue, are also important as they impact on quality of life, daily living activities and may result in mood disturbance [11]. High symptom burden is also a known risk factor for adverse psychological adjustment in people with cancer [13, 14].

People with haematological cancers receive chemotherapy in either day treatment units or in-patient settings, followed by discharge into the community. Patients are required to monitor their side-effects at home but may be reluctant to inform their treatment team when problems arise [15]. Structured symptom assessment tools and the implementation of timely management strategies improve patient physical and emotional outcomes [16–18], but traditionally rely on retrospective patient recall (from their previous appointment or treatment), are prone to recall bias [19] and impede timely clinical responses. The ability to capture patient-reported symptom data in real-time is, therefore, the ‘gold standard’ to allow rapid clinical decision-making and interventions to improve patient outcomes.

### Need for patient self-management of side-effects and the role of the specialist nurse

Patients discharged into the community setting following each chemotherapy treatment cycle are also required to monitor their health and engage in self-care activities to prevent, or reduce, the severity of numerous, and possibly complex side-effects. In addition, patients often report the need ‘to be informed about the things you can do to help yourself get well’ [20]. Developing patients’ self-care skills is critically important to ensure safe and high quality care at home. Previous studies examining the nurse’s role in promoting patient self-care demonstrate the potential of nurses to reduce psychological distress [13], reduce patient concerns about treatment [13], decrease barriers to self-care [21] and improve symptoms [13] using short duration nurse-led interventions. Various strategies have also been used to boost these interventions including, evidence-based messages via DVD [13], or telephone follow-up, in addition to face-to-face sessions [13, 21]. Despite positive results, these interventions were hampered by the inability to respond to patient concerns in real-time. Systematic reviews in cancer [22], and chronic disease [23], conclude that research, in particular intervention studies, are a priority to guide practice to improve patient self-management.

### Telehealth systems can improve patient outcomes

The International Organisation for Standardisation defines Telehealth as the ‘Use of telecommunication techniques for the purpose of providing telemedicine, medical education and health education over a distance’ [24]. More specifically this relates to the real-time remote exchange of physiological or symptom data between patients in the community and clinicians within a treatment facility and includes: the Internet; phone lines (land or mobile); or video links. To date, technology has enabled successful assessment of symptoms in patients with chronic disease resulting in improved patient outcomes and decreased hospital stays/health system costs [25–32]. However, few studies report Telehealth systems for cancer patients and most only focus on acceptability, feasibility or useability data [33]. Only five papers to date attempt to quantify benefits in patients with cancer (eg during chemotherapy treatment; post-surgery) which include: decreases in fatigue, pain, depression, post-operative symptom threshold events; interference with activities of daily living and; preventable use of health care services (eg clinic visits, bed days of care) [34–38]. Despite the promising early findings, all Telehealth research in cancer to date has serious limitations including sub-optimal study design (insufficient power, no record of patient adherence to self-care/intervention adherence) or health economic analyses [33]. In addition, no cancer Telehealth systems have incorporated explicit coaching of patients in the self-care advice delivered; or have been developed for use with high-risk clinical groups or within an Australian context [33].

### Development of a remote monitoring system for haematological cancer patients

Given the early success of the Advanced Symptom Management System (ASyMS) developed in the UK by Kearney and colleagues, a remote-monitoring system used in a range of patient settings [36, 39–42] a collaboration to develop a remote monitoring system specifically for Australian patients with haematological cancers (i.e. those at increased risk of chemotherapy toxicities) was undertaken. ASyMS uses a touch screen mobile phone application to collect daily data about patient chemotherapy side-effects. Side-effects which exceed pre-determined limits (as assessed by underlying software algorithms) create alerts in real-time to the hospital treatment team who would log into the secure system website, review patient data and contact the patient for further assessment/clinical intervention. The application also provided tailored self-care for patients reporting side-effects alongside plotting side-effect data over time.

Content for the Australian system to be utilised by haematological cancer patients (i.e. side-effects monitored, evidence-based self-care advice and alerting algorithms) was developed following a review of the literature and extensive consultation with haematology nurses, clinicians and patients. The system was piloted and found to be highly acceptable to both patients and clinicians with feedback around content and functionality used to improve the system [43]. However, it was noted that some patients required additional/ongoing support to utilise the full functionality of the remote monitoring system; to understand that system alerts were expected from patients receiving treatment and; that patients should not feel uncomfortable or guilty about triggering these alerts [43].

### Value adding to remote side-effect monitoring

It is important that Telehealth systems evolve to combine ongoing human contact (eg nurse coaching/support) with technology delivered care, rather than relying on the technology alone. This means that a remote monitoring system is only *part* of a structured patient intervention. A vital addition to ASyMS in the Australian system is an evidence-based nursing intervention, including patient education and coaching in self-care. Coaching patients in self-care is associated with improved patient outcomes/decreased psychological distress [22, 23] and previous studies have identified that nurse-led interventions focusing on patient education and self-care coaching can improve patient outcomes [13, 21]. *However*, *these study results were limited by not being able to respond to patient side*-*effects in real-time*. Telehealth, therefore, have the potential to influence supportive care outcomes by assisting nurses to respond to a patient’s *actual* symptoms with evidenced-based self-care, rather than preparing patients for *potential* side-effects which may never occur.

We have developed a dual intervention, the Patient Remote Intervention and Symptom Management System (PRISMS) which combines a remote monitoring system based on the ASyMS system developed by the authors [36, 43] with an evidence-based nurse-led intervention to improve patient outcomes during chemotherapy. Ongoing monitoring and clinical response to patient alerts will, therefore, be accompanied by scheduled nurse follow-ups to ensure appropriate: usage of system functionality by patients, uptake of self-care behaviours and normalisation/reassurance that symptom alerts are expected and part of the nurses role. This intervention was previously successfully trialled in a small Phase II trial from which the methods remain largely unchanged.

This Phase III multi-site randomised controlled trial will determine the effectiveness of the PRISMS intervention to improve patient physical chemotherapy related side-effects and psychological health, alongside a cost-effectiveness analysis. This protocol describes the methods proposed in the conduct of the Phase III trial which is set within the Medical Research Council (MRC) framework for evaluating complex interventions [44].

## Methods/Design

This study is a multi-site, parallel group, multi-site Phase III randomised controlled trial. Eligible participants will be randomised in a stratified 1:1 allocation ratio based on factors such as cancer type to one of two arms: an intervention arm in which the participants receive the PRISMS remote symptom monitoring nurse-led intervention *in addition* to their Usual Care and a control arm in which they receive only Usual Care.

### Study setting and participants

Patients will be recruited from two institutions located in two capital cities of two different states of Australia.

#### Patient inclusion criteria

Patients will be eligible to participate in the trial if they have: a confirmed diagnosis of Hodgkin’s lymphoma, non-Hodgkin’s lymphoma (NHL) or chronic lymphocytic leukaemia (CLL); are scheduled to receive at least 2 cycles of chemotherapy; are aged 18 years and over; and are able to provide informed consent prior to commencing chemotherapy treatment.

#### Exclusion criteria

Patients are ineligible to participate if they: are unable to read/write/speak English sufficiently to complete study measures; are unable to complete the study measures due to a pre-existing disability; have an Eastern Cooperative Oncology Group (ECOG) status of 3 or greater; have severe cognitive or emotional issues (as assessed by the treatment team) which could limit the completion of the study measures or; are participating in a conflicting drug trial such as a non-standard or trial chemotherapy agent.

#### Intervention nurse selection

Senior cancer nurses from the Chemotherapy Units at both sites will be selected by Nursing Unit Managers and trained to deliver the intervention/respond to PRISMS alerts during business hours. All senior nurses in the role of Patient Service Manager at the Melbourne site will be trained to respond to PRISMS alerts after hours. At the Brisbane site no nurses will be trained to respond to after-hours alerts but rather patient alerts will be directed to the consulting haematologist to fit in with Usual Care at this site.

### Recruitment

The following recruitment processes will be tailored in consultation with staff from each site, to ensure that they comply with existing clinical procedures and systems. Data managers (DM) based at each site will identify eligible patients from the haematological outpatient and chemotherapy treatment lists, with the assistance of haematology clinicians, nurse coordinators and senior ward staff. Eligibility will be confirmed with a member of the treating team prior to approaching any patients to clarify any details from the medical records, and to ensure the team is aware of the patient’s involvement with the study. If a patient is considered too unwell by his/her treatment team to participate in the study at the time of initial screening, the patient will be excluded from participation and will not be re-approached at a later date. No further action will be required from the treatment team.

#### Recruitment from clinical areas

The DM will approach eligible patients at a convenient time before or after a medical appointment. The study will be explained verbally and a copy of the Participant Information and Consent Form will be provided. Patients will be reminded that participation in the study is voluntary and given the opportunity to ask questions. They may take the information home for further consideration. People who would like to participate will be asked to sign the consent form (ie to provide written informed consent for study participation). Those who decline will be asked for verbal consent to collect basic demographic and clinical information (including age, sex, diagnosis and chemotherapy protocol) from their records to examine potential recruitment bias. Reasons for refusal will be recorded when provided and no further contact will be made with these patients. Patients who consent to study participation will be given the Baseline Questionnaire to complete prior to randomisation.

#### Recruitment via telephone

In the case where patients do not have any clinic visits scheduled immediately prior to commencement of chemotherapy, a DM will contact the patient by phone following consent to approach being given by a member of the treatment team. The DM will explain the study verbally and ask for patient consent to post more information about the study to the patient (i.e. the study consent form and a postage paid return envelope) and organise a time to call the patient back to address any questions they may have. People who would like to participate will be asked to sign the consent form. Following receipt of a signed consent form, the DM will mail a copy of the Baseline Questionnaire with a postage paid return envelope. Patients will be asked to complete the questionnaire and mail it back to the study.

### Registration and randomisation

Following consent and completion of the Baseline Questionnaire, the DM at the coordinating site will register the participant, confirming all eligibility/registration details and will allocate a participant identification number. The participant will be randomised by the DM using a centralised randomisation database 1:1 to either the control or intervention groups. Participants will be stratified according to treating hospital, cancer type, treatment toxicity (high versus low as predetermined by clinical investigators based on the likelihood of patients developing febrile neutropenia during treatment) and receipt of previous chemotherapy treatment (received no previous chemotherapy versus received some form of previous chemotherapy either orally or intravenously). Minimisation will be used to balance randomisation across strata (also known as baseline adaptive allocation) – this is a dynamic allocation process where the probability of a patient being allocated to a particular arm varies for each patient being randomised, depending on the patients who have previously been randomised in the study and their status at the stratification factors [45, 46]. No sequence is generated in advance, instead the randomisation database generates a set of probabilities the particular patient will be allocated to an arm, based on the above set of pre-specified rules. The minimisation process aims to allocate equal numbers of patients to each arm simultaneously over many strata. The multiple strata are combined into a score for the degree of imbalance between arms, and then the allocation is made based on a coin algorithm.

Participants randomised to the intervention arm will be scheduled for their first session with an intervention nurse for education and training in the use of the Android tablet.

### Data collection

#### Patients

Baseline data will be collected prior to the first chemotherapy treatment cycle. Follow-up data will be collected at 7 time points:

- At completion of chemotherapy treatment cycle 1 (Follow-up 1)
- Mid-cycle following of second, third and fourth chemotherapy cycles (Follow-ups 2a, 3a and 4a)
- At completion of chemotherapy treatment cycles 2, 3 and 4 (Follow-ups 2b, 3b and 4b)

**Fig. 1:**
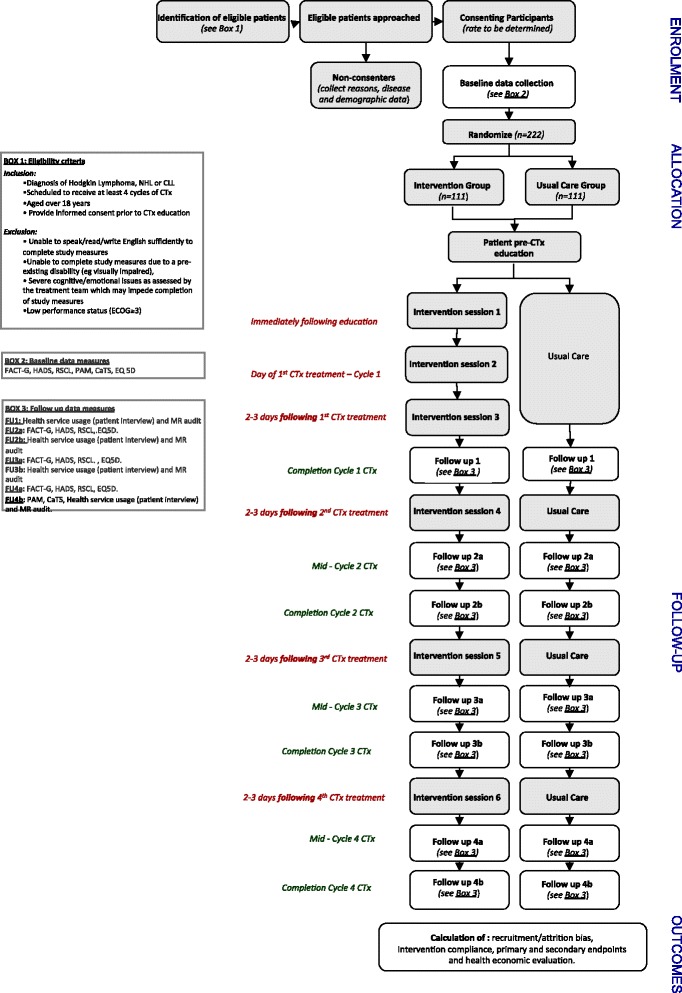
Flow diagram of study design

The questionnaires completed at each study time point are summarised in Fig. 1. Data collection has been split across 2 time periods in each treatment cycle so as to allow the capture of side-effect and related outcomes at a point in the cycle where they are likely to be problematic (i.e. Follow-ups 2a, 3a and 4a) as the timeframe for each of the validated outcome measures is within the past week (i.e. Follow-ups 2a, 3a and 4a). Other measures, such as health service usage rely on data from an entire cycle and are, therefore, measured at the conclusion of chemotherapy treatment (CTx) cycles (i.e. Follow-ups 2b, 3b and 4b).

Follow-ups 2a, 3a and 4a will be self-completed by the patient at home. Patients will be mailed copies of the questionnaires (just prior to the questionnaire completion due date) along with a postage paid envelope. The date to complete the questionnaires will be written on the cover page and a reminder phone call on the due date completed by the DM. Patients will be asked to complete the questionnaires and return them in the envelope provided. Follow-ups 1, 2b, 3b and 4b part 1 and 2 data will be completed by patients and DM together immediately prior to their scheduled appointments for chemotherapy cycles 2–5.

### Measures

#### Pen-and-paper measures

1. Demographic Questionnaire (baseline only) will be used to assess basic patient demographic information including: current employment status, occupation, level of education, living arrangements and number/age of children, country of birth, languages spoken) and previous use of technology (including smart phones, computers and the Internet).
2. Rotterdam Symptom Checklist (RSCL) will be used to assess patient symptom burden. The 30-item checklist is appropriate for use in mixed cancer populations including those undergoing chemotherapy, radiotherapy and/or surgery [47]. Participants use a four-point Likert-type scale to rate the extent to which they have been bothered by each symptom during the past week. Item scores can be used to compute three scales including a physical symptom distress level, psychological distress level and activity level impairment. All scales demonstrate acceptable internal consistency (alpha = 0.71–0.90) and good convergent (*r* > 0.58) and discriminative validity [47] and sensitivity to change in a longitudinal setting [48].
3. Functional Assessment of Cancer Treatment – General scale (FACT-G) questionnaire will be used to assess patient health-related quality of life (QOL). The 27-item FACT-G assesses four specific domains including physical, social, emotional and functional well-being [49]. The scaling and unidimensionality of its subscales have been broadly confirmed by both factor and Rasch analysis respectively [50]. FACT-G subscales demonstrate acceptable internal consistency (alpha = 0.72–0.90), good convergent (*r* > 0.51), divergent (*r* < 0.22) and discriminative validity [49] and responsiveness to psychosocial interventions [51].
4. Hospital Anxiety and Depression Scale (HADS) will be used to measure psychological distress [52]. The 14 items of the HADS form a uni-dimensional construct of psychological distress [53]. HADS has demonstrated high internal consistency (alpha = 0.85) in cancer patients [53, 54] and responsiveness to psychosocial interventions [55].
5. EuroQol-5D (EQ-5D) descriptive system and visual analogue scale will be used to assess health utility/QOL. The EQ-5D is a standardised measure [56] of health status developed specifically for economic evaluation. In cancer patients it has acceptable convergent validity (*r* > 0.49), internal consistency (alpha = 0.71), test-retest reliability (kappa = 0.7), discriminative validity and sensitivity to change in a longitudinal setting [57, 58].
6. Patient Activation Measure (PAM; baseline and end of cycle 4 only) will be used to assess patient activation (i.e. ability to self-manage health). The 13 items of the PAM form a unidimensional construct of knowledge, skills and confidence for self-management. It has demonstrated high internal consistency (alpha = 0.90) and excellent discriminative validity based on known group comparisons [59].
7. Cancer Treatment Scale (CaTS; baseline and end of cycle 4 only) will be used to assess cancer treatment-related information and support needs. This 25 item measure forms two subscales: sensory-psychological concerns and procedural concerns. Both subscales demonstrated high internal consistency (alpha > 0.90) and good divergent validity (with the HADS: *r* < 0.26) [60]. Both subscales were sensitive to change in a recent intervention study [13].

#### Medical record audit

Demographic and clinical data collected from the patient medical record will include: age, gender, stage of disease; length of time since diagnosis; treatment received in past; ECOG performance status, medications and chemotherapy treatment regimen. Usage of hospital resources will be undertaken via medical record audit to assess emergency room presentations, unplanned hospital admissions, total bed days of care. Use of nursing time, allied health services, clinical investigations/procedures and other related staff time and resource use by patients in both conditions will be recorded. Changes in chemotherapy treatment dose including dose reductions, and treatment delays will also be recorded.

#### Structured costs interview

Use of other health services (and patient-related costs) not recorded in the primary patient medical record, will be assessed through the use of the Patient Health Services and Costs Interview. This interview is conducted with the patient by the DM to identify which health- related services were utilised and any medications prescribed/used in the previous cycle in order to assign costs.

#### External hospital data

In the case where DMs identify that a patient has been treated and/or admitted to a hospital which is *not* the primary treating hospital from the medical record audit or costs interview then a letter to this external treating hospital will be sent requesting basic details.

#### PRISMS electronic log data

Data from the PRISMS log files will be utilised to assess patient compliance with daily symptom reporting and reported use of self care activities. Log data will also be used to assess nursing compliance with the system including: timely response to alerts; documentation of actions taken in response to alerts and completion of checklists documenting scheduled interventions.

### The PRISMS intervention

The PRISMS intervention is guided by 5 principles which are linked to improved patient outcomes including: 1) *promotion of patient involvement and engagement* [13, 61]; 2) *tailoring to specific need* [62]; 3) *emphasising evidence based self-management* [13, 63–67]; 4) *involvement of significant others* [68] *and*; 5) *preparing patients for treatment and potentially threatening medical procedures* [13, 69–71].

The PRISMS intervention will include two synergistically operating parts: 1) *a mobile phone-based remote monitoring system* with built in patient self-care advice and clinician alerting algorithms [43]; and 2) *a structured nursing support intervention relating* to: patient use of the remote monitoring system; patient coaching in self-care strategies and *ongoing* access to 24-hour nursing support/clinical interventions, triggered by real-time system alerts.

#### The mobile phone enabled remote monitoring system

**Fig. 2:**
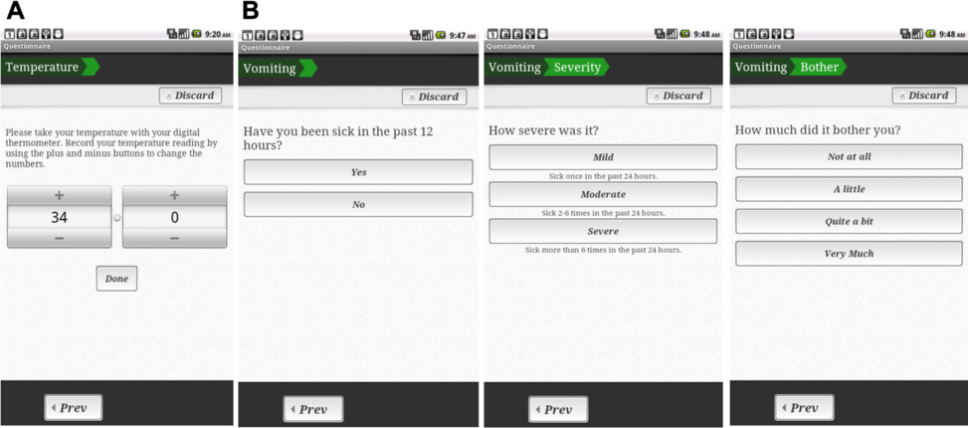
Screenshots from the Patient Remote Intervention and Symptom Management System (PRISMS) patient remote monitoring application illustrating: **a** Temperature input. **b** Assessment of vomiting

This system is based on the prototype previously developed and piloted [43] and based on the ASyMS system developed by Kearney et al. [36]. Patients are provided with a 7″ Android tablet which has the remote monitoring software application installed. Patients are trained in the use of the remote monitoring system by nurses. The Android application prompts patients twice daily to complete a side-effect assessment questionnaire by following instructions on the touch screen (Fig. 2). Patients can also complete questionnaires at anytime they feel unwell. The questions about common chemotherapy related side-effects are based on the Chemotherapy Symptom Assessment Scale [72] which measures both severity (mild, moderate, severe) and distress (not at all, a little, quite a bit, very much) caused by symptoms. Symptom severity *descriptors* were adapted from the Common Toxicity criteria for Adverse Events (CTCAE V4.0) [73] for consistency in interpretation between patients and clinical grading of symptoms by clinicians. Side-effects monitored include: rigors, bleeding, nausea, vomiting, diarrhoea, mucositis, fatigue, constipation and peripheral neuropathy. Patients are also prompted to rate their ability to complete daily living activities [73] and to input additional parameters such as body temperature, food intake and fluid intake into the system. Once weekly, patients also answer questions about levels of anxiety, depression and psychological distress as well as their use of self-care behaviours to alleviate side-effects. At the completion of each patient questionnaire the Android application automatically uploads and encrypts all patient questionnaire data via General Packet Radio Services to a secure study server.

**Fig. 3:**
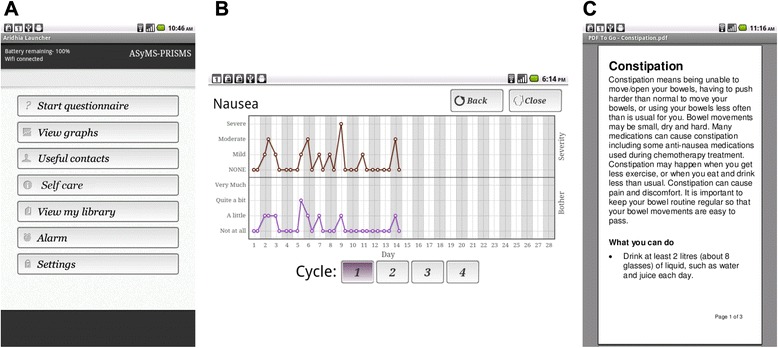
Screenshots from the Patient Remote Intervention and Symptom Management System (PRISMS) patient remote monitoring device showing: **a** The main menu. **b** A graph illustrating the severity and bother due to nausea. **c** An excerpt of self-care information about constipation

The report of any side-effects by patients completing the questionnaire triggers the provision of tailored evidence-based self-care advice to the patient (Fig. 3). The self-care advice was developed from a systematic review of the literature [74] and in consultation with haematology clinicians and patient advisors, and tested in our previous pilot study [43]. The self-care advice consists of simple instructions to self-manage side-effects. The patient tablet application also contains a graphing function for patients to view their side-effects profiles over time and an extensive library of written, audio and video information resources (Fig. 3).

##### Clinician alerts

The server software which receives patient data contains in-built decision support algorithms which act to trigger Email alerts to a dedicated mobile phone held by nurses. Alerts are divided into two categories: red – for life-threatening or urgent issues (eg fever) which must be addressed within 15 minutes and; amber – for moderate/non-life-threatening issues which should be addressed within 8 hours. Alerts are based on those developed from a review of the literature, extensive clinician consultation and then subsequently piloted and refined in a previous pilot study [42]. Alerts are based on either the severity and/or distress relating to reported side-effects. In addition, patients who have not completed side-effect questionnaires for > 24 hours will also generate an alert to nurses known as a ‘missing alert’ which also requires follow-up within 8 hours.

**Fig. 4:**
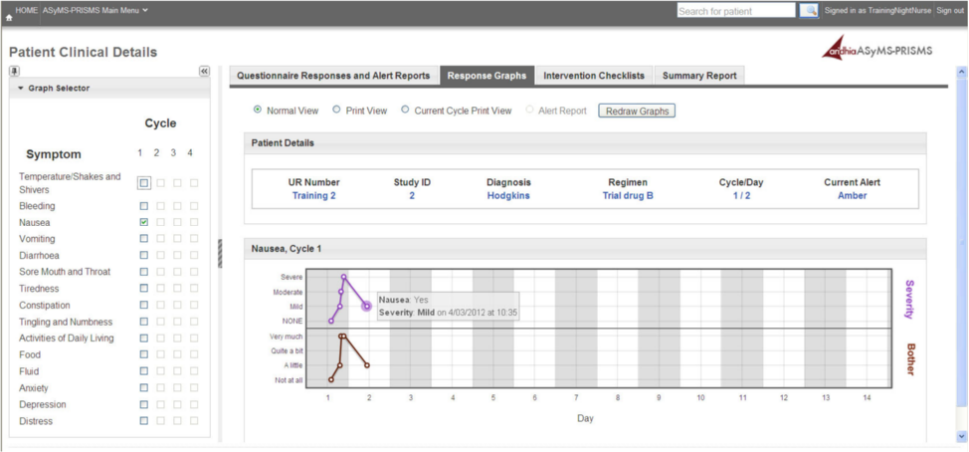
Screenshot from the Patient Remote Intervention and Symptom Management System (PRISMS) clinician website illustrating a graphical summary of a patient temperature profile with graph selector menu

**Fig. 5:**
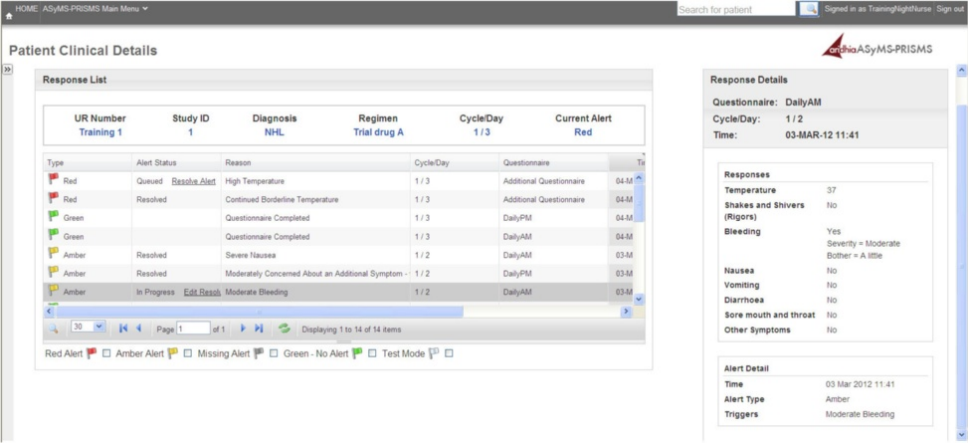
Screenshot from the Patient Remote Intervention and Symptom Management System (PRISMS) clinician website illustrating history of individual patient alerts and side-effects reported

Upon receipt of alerts, intervention nurses will access a secure website to view the latest patient questionnaire data. A history of patient symptoms across cycles is provided in both tabular and graphical formats to quickly orient the nurses to the patient’s situation (Figs. 4 and 5). Trained nurses contact the patient by phone to undertake further assessment and management. Evidence-based triage and assessment algorithms are also provided on the secure website alongside evidence-based self-care information to direct nursing care. Intervention nurses record all actions in a series of open fields and drop down boxes on the secure website. Monitoring of patients (via the SMS alerting system) occurs 24 hours/day.

#### Structured nursing support enabling self-management

**Fig. 6:**
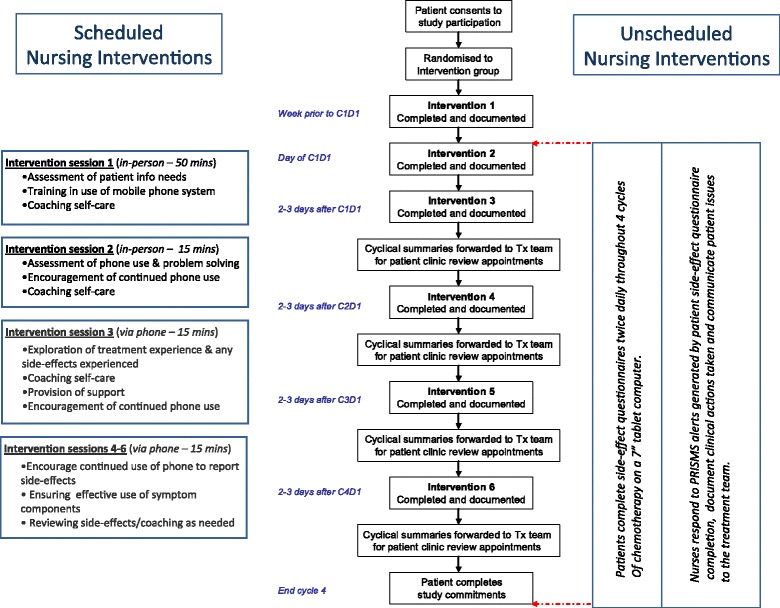
Summary flow diagram of the Patient Remote Intervention and Symptom Management System (PRISM) intervention (Abbreviations: C1D1, Day 1 of chemotherapy treatment cycle 1; C2D1, Day 1 of chemotherapy treatment cycle 2; C3D1, Day 1 of chemotherapy treatment cycle 3; C4D1, Day  1 of chemotherapy treatment cycle 4)

The structured nursing support component of this intervention will include 6 scheduled face-to-face or phone intervention sessions (Fig. 6).

- *Intervention Session 1:* this 50-minute face-to-face session occurs directly following pre-chemotherapy education and incorporates 4 components: i) training in the use of the mobile phone remote monitoring system; ii) eliciting and responding to patient’s key concerns; iii) provision of evidence-based side-effect management strategies and; iv) coaching in self-care advice for most frequent side-effects. Coaching involves reinforcing the importance of patients communicating about their side-effects with the treatment team; identifying potential barriers to self-care, reinforcing the benefits of self-care interventions, assisting the patient to develop plans to overcome barriers to self-care action and where appropriate encouraging the involvement of the patient’s family in self-management [60–62].
- *Intervention Sessions 2–6* : Intervention Sessions 2 (15-minute face-to-face) and 3–6 (15-minute via telephone) focus on answering additional patient questions about treatment or use of the system, coaching in use of self-care and utilisation of the full functionality of the PRISMS tablet computer application (Fig. 6).

### Quality control

#### Training intervention nurses

At each site specialist cancer nurses will be trained to provide scheduled patient interventions during business hours and to respond to real-time patient generated system alerts. Training will take place in 2 sessions of 4 hours and consists of: a project overview; developing familiarity and skills in the use of the remote monitoring/management system and responding to system alerts; communication skills training for in eliciting and responding to emotional cues; coaching in self-care strategies and; role-play scenarios with simulated patients. A detailed intervention manual containing all aspects of the training as well as evidence-based self-care algorithms will also be provided to all intervention nurses.

#### After hours nurses

After hours nurses will be trained in how to respond to real-time patient generated system alerts using the website. A short manual containing instructions on how to respond to alerts and use the website will be provided.

#### Data manager training

DMs with experience in participant recruitment and coordinating research projects will be trained to identify eligible patients, liaise with hospital staff and intervention nurses, approach patients, randomise consenting patients, administer pen-and-paper outcome measures and interviews at all data collection time points, manage participant databases, and report data back to the project team.

#### Quality assurance of the intervention

All scheduled face-to-face and phone interventions will be digitally recorded for assessment of content and adherence to the intervention manual. Intervention nurses will also be required to record all interactions and interventions within the PRISMS website from drop down menus/open text boxes. Completeness of intervention delivery can then be assessed for adherence to the intervention manual with data from both the recorded conversations and data entered into the website. The time taken for intervention nurses to respond to red/amber alerts will be recorded automatically by the website to ensure that alerts are responded to within designated time frames. All nurses involved in the study, will have access to evidence-based algorithms/self-care strategies for managing patient side-effects on the system. Nursing responses to real-time patient alerts documented on the system will be assessed for compliance with triage algorithms provided.

Patient adherence to daily completion of side-effect questionnaires with the Android application will be assessed for timeliness and patient *use* of the prescribed self-care information provided by the Android application will also be monitored with a weekly self-report questionnaire.

#### Diffusion of the intervention

Diffusion of an intervention relates to how a novel idea or practice spreads through a population – for example the adoption of a new behaviour. In the case of an intervention study such as PRISMS, it is possible that nurses trained to deliver the PRISMS intervention may discuss novel practices with nurses delivering Usual Care; thus changing Usual Care and diluting the potential overall intervention effect. In a previous study of a pre-CTx education intervention [13] we successfully trained a subgroup of Day Chemotherapy Unit nurses for intervention delivery. A random analysis of recordings from both control and intervention group education sessions subsequently showed *no evidence of diffusion over time*. In this study diffusion will be minimised by: 1) instructing intervention nurses about the effects of diffusion/impact on study outcomes; 2) intervention nurses not treating control group patients and; 3) control group patients not using the remote monitoring system thereby removing the alerts which structure a large component of the intervention.

Nurses who monitor real-time alerts after hours will not be able to avoid potentially providing care to control group patients who may ring the switchboard for information/clinical advice. However, when these nurses manage control group patients they will not have ready access to the evidence-based triage algorithms/self-care information located on the system website or data from side-effect history profiles available to intervention patients.

### Standard of care

#### Control group

Patients allocated to the control group will receive the following resources from the DM following randomisation: an evidence-based patient preparation for chemotherapy DVD [13]; a standard booklet ‘*Introduction to Chemotherapy*’; and standard drug information sheets. Control group patients will receive pre-chemotherapy education as per Usual Care at each site. They will also have routine access to the haematology treatment team at each site via: i) scheduled clinic visits, and ii) via hospital switchboards at other times.

#### Intervention group

Patients allocated to the intervention condition will receive standard care as described above in the control group. *In addition* they will also receive the PRISMS intervention.

#### Safety of the intervention

The intervention *is not* designed to replace normal patient contact with the hospital (i.e. Usual Care) but to enhance the current Usual Care. The patient Android application comes equipped with emergency and hospital phone numbers for patient reference and the tablet computer provided can also be used as a phone to contact the hospital when required. Should an error occur in the transmission of patient side-effect data from the patient tablet to the system server/website the software application will advise the patient of the failure of data transmission and, based on alerting algorithms, provide detailed instructions on who and when to call. The server containing the system is monitored daily for performance and any faults addressed by technicians.

### Outcomes

#### Primary hypothesis

Compared to the Usual Care control group, participants receiving the PRISMS intervention will report lower scores item scores for nausea, mucositis, constipation and fatigue of at least 0.4 standard deviations at follow-up (mid-cycle 2 of chemotherapy treatment).

#### Secondary hypotheses

*Specific secondary hypotheses are that:*

1. Participants receiving the PRISMS intervention will report lower scores on item scores for nausea, mucositis, constipation and fatigue compared to the Usual Care control group at follow-up (mid-cycles 3 and 4);
2. Participants receiving the PRISMS intervention will report lower item scores for vomiting and diarrhoea compared to the Usual Care control group at follow-up (mid-cycles 2, 3 and 4);
3. Participants receiving the PRISMS intervention will report lower levels of psychological distress, better quality of life, higher levels of patient activation (ability to self-manage health) and lower levels of cancer information and support needs compared to the Usual Care control group at follow-up (mid-cycles 2, 3 and 4); and
4. The PRISMS intervention will be cost-effective compared to the Usual Care comparator at follow-up (end of chemotherapy cycles 2, 3, and 4).

Primary and secondary hypotheses for nausea, mucositis, constipation and fatigue differ only in the predicted chemotherapy cycle. This is based on previous qualitative data relating to the PRISMS intervention which suggested that patients find the system to have greatest value in cycles 1 and 2 as well as the issue that not all patients who are originally scheduled to receive 4 cycles of treatment go on to complete all cycles.

### Sample size

Primary outcomes are nausea, mucositis, constipation and fatigue as assessed by the RSCL items labelled ‘nausea’, ‘sore mouth/difficulty swallowing’, ‘constipation’ and ‘tiredness’ respectively. All items have a possible scale range of 1 to 4 (corresponding to a rating of ‘not at all’, ‘a little’, ‘quite a bit’ and ‘very much’ respectively) with higher scores indicating a higher levels of burden or impairment. These outcomes were chosen because they are common in patients receiving chemotherapy, are targeted by the PRISMS intervention and have demonstrated improvement following Telehealth interventions [33].

Sample size calculations were based on 80 % power, a 2-sided independent-samples *t* test with an alpha level of 0.05 and a standardised effect size of 0.4. In the absence of minimal important difference estimates for the RSCL, or any other symptom assessment tool validated for use in cancer patients, evidence-based effect sizes for the EORTC QLQ-C30 symptom scales and individual items were used as a guide [75, 76]. In this case, for all QLQ-C30 scales/items excepting the dyspnoea item, a standardised effect of 0.4 represents the lower threshold of a medium-sized clinically relevant difference between groups of cancer patients. Given these specifications, a total of 200 patients (100 per arm) is required. Assuming attrition of up to 10 %, a total of 222 patients (111 per arm) are needed at baseline.

### Recruitment

Based upon recruitment/retention statistics collected in the Phase II trial at the Melbourne site in 2012–13, and upon the predicted eligible patient population presenting at the Brisbane site, we estimate that around 108 patients will be recruited in 12 months across the 2 sites with total recruitment time of approximately 25 months.

### Analyses

#### Quantitative

All data will be entered into Microsoft Excel 2003 (or higher) (Microsoft Inc., Redmond, WA, USA) then imported into SPSS Windows Version 21 (or higher) (SPSS, Chicago, IL, USA) for scoring and analysis. R version 3.0.1 (or higher) and the R package ‘ggplot2’ will be used to prepare all graphs and plots [77].

Prior to formal analysis, descriptive statistics and graphical displays will be used to identify missing and out-of-range values, assess the plausibility of means and standard deviations (SDs) for all variables and identify outliers and screen continuous variables for normality.

#### Recruitment bias

Recruitment bias will be assessed by comparing demographic and clinical variables of patients who consent to participate and those who decline participation using *t* tests (or Mann-Whitney *U*) and chi-squared (or Fisher’s exact) tests as appropriate.

#### Differential attrition

Possible differential attrition will be assessed by comparing baseline characteristics of drop-outs and continuing participants using *t* tests (or Mann-Whitney *U* tests) and chi-squared (or Fisher’s exact) tests as appropriate.

#### Outcome analyses

Analyses will be by intention-to-treat. Primary and secondary outcome analyses will be carried out by fitting a linear mixed model to each outcome separately using all available data. A cell mean model (suppressing the default intercept) will be used to estimate mean scores for each group (study arm) by time combination; all models will include a fixed group by time effect and no random effects [78]. An unstructured covariance type will be used to model the covariance structure among repeated measures and all models will be estimated by maximum likelihood. The primary analysis will be group comparisons at post-baseline time points; these will be performed using contrasts within the proposed models. The group by time interaction will be used to assess the overall pattern of change [79]. A ‘toxicity’ by group by time interaction will be added to each model to assess whether participants receiving chemotherapy protocols classified as either ‘low-toxicity’ or ‘high toxicity’ respond differently to the PRISMS intervention. Adjusted analysis including using baseline responses to study measures and the remaining stratification factors will also be investigated.

After inspection of the data, the appropriateness of suggested methods will be assessed and revised as necessary. Specific emphasis will be placed on assessing models proposed for the analysis of primary and secondary outcomes that are measured on an ordinal scale; that is, nausea, mucositis, constipation and fatigue as assessed by the RSCL. When applied to ordinal outcomes, models for continuous outcomes can yield biased estimates of regression coefficients. When the distribution of scores on ordinal variables is highly skewed, this bias may be large and the use of methods specifically designed for ordered data is recommended [80]. In this case, if graphical exploration of the data and/or regression diagnostics indicates violation of model assumptions, relevant analyses will be carried out by fitting an ordinal logistic generalised linear mixed model to each outcome separately using methods described by Hedeker [81]. In brief, fully parameterised models will include fixed effects for group, time plus a group by time interaction, as well as random subject and time effects.

#### Exploratory analyses

Linear regression models will be used to explore predictors of health-related quality of life and psychological distress at baseline. Growth curve models will be used to explore associations between baseline characteristics and longitudinal outcome data in the control group. Linear regression models will also be used to explore predictors of health services utilisation.

#### Intervention compliance

Frequency statistics (i.e. raw counts and averages) will be used to summarise data relevant to patient/nurse intervention compliance. This data will come from two sources: the electronic log of the PRISMS system and digital recordings of face-to-face and phone intervention sessions.

#### PRISMS electronic log files

With respect to *patient data* this will include: frequency of < 2 symptom questionnaires being completed within a 24-hour period; Frequency of ‘missing alerts’ generated (i.e. no symptom questionnaires submitted within a 24-hour period); Frequency of missing weekly self-care use questionnaires.

With respect to *nursing data* this will include: frequency of red alerts responded to within designated timeframe (i.e. 15 minutes) as measured by login to secure server; frequency of amber alerts responded to within designated timeframe (i.e. 8 hours) as measured by login to secure server; frequency of missing alerts responded to within designated timeframe (i.e. 8 hours) as measured by login to secure server; frequency of documentation of actions taken in response to alerts and completion of checklists for scheduled interventions.

#### Digital recordings

A content checklist has been developed to assess ‘intervention completeness’ for each intervention time point. Checklists will be based on the nursing intervention manual as previously developed by the research team. A sub-set of recordings (20 %) will be listened to by 2 members of the research team and a percentage completeness figure calculated.

#### Economic evaluation

Cost utility analysis (a form of cost-effectiveness analysis), measures benefits in quality- adjusted life years (QALYS). This will allow for the comparison of efficiency of patients receiving the PRISMS intervention and those receiving Usual Care in terms of cost-per-QALY gained. Analyses will be undertaken both from a broad societal perspective in addition to a health care perspective.

The incremental cost of the intervention will be compared to the incremental health outcome improvement attributed to the intervention. Results will be expressed as cost per QALY gained (i.e. the incremental cost-effectiveness ratio (ICER) will be determined for patients receiving either the PRISMS intervention or Usual Care) within the trial period and projected beyond the trial period using appropriate economic modelling. Uncertainty in the cost and outcome data will be further evaluated via sensitivity analyses whereby key evaluation parameters (such as unit costs) are varied to assess the impact on study conclusions.

Economic measures for both benefits and costs utilised in these analyses include the EQ-5D as a utility measure of patient quality of life; the Health Services and Costs Questionnaire to determine use of health care services and medicines and; a medical record (MR) audit of both treating hospital records and any external hospital records (eg emergency admissions). More specifically, MR audits and the Health Services Usage and Costs Questionnaire will document: emergency room presentations, planned/unplanned hospital admissions, total bed days of care and reasons of hospitalisation, use of nursing/allied health resources, clinical investigations, medications prescribed (including chemotherapy and dose reductions and treatment delays), use of community health resources (eg general practitioners (GPs), allied health), use of alternative therapies, community services (eg meals on wheels); use of transport; days out of role (paid/unpaid) for both patients and carers, alongside any other costs incurred to patients/carers as a result of their cancer treatment. Data collection will cover both that paid for by the individual (before/without rebate) and also that paid for by third parties (eg rebated dollar amount by Medicare or private health insurance companies). Calculation of the cost of the PRISMS intervention itself will include only the costs of the intervention delivery (i.e. excluding development or research costs). Research team records (eg PRISMS website; data transmission costs, training time/materials) will be used to determine the cost of the intervention. Measured resource use will be valued using existing unit costs such as the National Hospital Cost Data Collection-Cost Report; Australian Refined Diagnosis Related Groups for inpatient health costs, Medicare Benefits Schedule for outpatient costs, Pharmaceutical Benefit Scheme for pharmaceutical costs and Australian Bureau of Statistics for estimates of Australian earnings for productivity effects.

### Ethical issues

The study protocol has been ethically approved by institutional ethical review committees at both participating sites (Peter MacCallum Cancer Centre Human Research Ethics Committee and Bellberry Human Research Ethics Committee for ICON Cancer Care). The rights and welfare of the participants will be protected according to the National Health and Medical Research Council of Australia’s National Statement on Ethical Conduct in Human Research. Clinical care and emergency medical services are provided by participating institutions. Data that is collected as part of the study will not be linked to any individual.

## Discussion

The proposed system combines cutting edge technology with systematic evidence-based, nursing consultation. It is internationally innovative and tailored to the real-time monitoring of CTx toxicities and the provision of tailored self-care to address individual needs. Given the aging of the Australian population and the consequent expected increase in people diagnosed with cancer, it is imperative to find cost-effective strategies to improve the quality and safety of cancer care in the community whilst improving patient outcomes. These systems are most critical where CTx treatment has a high-risk of toxicity and potentially life-threatening consequences leading to unplanned hospitalisations, such as with haematological malignancies, including leukaemia and lymphoma. The development of Telehealth systems and interventions which allow for the real-time remote monitoring and management of high risk patients during treatment are, therefore, a priority.

### Methodological rigour

This study will be the first technology-mediated nurse-led intervention study for cancer patients that meets the Consolidated Standards of Reporting Trials (CONSORT) criteria for randomised controlled trials [82] and fits within the MRC framework for the analysis of complex interventions [44]. Participants will be randomly allocated by a computer after eligibility assessment and the collection of baseline data. Intervention procedures will be strictly monitored for protocol adherence. The relatively short follow-up period will minimise dropout. Reasons for attrition will be recorded, and recruitment and dropout bias assessed. Blinding of patients and providers cannot be achieved with this study design. However, outcome assessment will be by patient self-report questionnaire, obviating the need for researcher blinding. A previous shortcoming of similar research [33] is the lack of knowledge of patient adherence to completing self-care behaviours when interpreting study outcomes. This system is, therefore, also unique in its ability to regularly measure patient adherence to self-care behaviours for key side-effects. Quantitative assessment of both nursing and patient intervention compliance will also be invaluable in guiding system or intervention changes needed to maximise system useability and intervention compliance for future studies.

As the intervention has been grounded in the current nursing models of patient care, alongside the development of detailed study training materials, the roll-out of the intervention to routine clinical practice following a successful Phase III trial could also be completed relatively efficaciously. The suitability of the system for both in-patient and out-patient treatment populations also makes it widely applicable to a range of chemotherapy treatment protocols.

The PRISMS intervention is innovative and tailored to the real-time monitoring of chemotherapy toxicities and the provision of tailored self-care to address individual needs. It is the first system designed to specifically monitor adult patients with haematological cancers undergoing chemotherapy. It is also the first randomised controlled trial of any Telehealth- mediated nurse-led intervention for cancer patients and builds from earlier ASyMS work and will, in tandem with the current large European (>1000 patients) ASyMS randomised controlled trial, add to our growing knowledge of the impact of these interventions on patient health outcomes and health system costs. Nursing interventions, combined with remote monitoring systems, such as those described in this protocol, are, therefore, likely to enable both more accurate symptom monitoring and management, combined with greater efficiency and effectiveness of nursing roles in haematology.

## Trial status

This trial is currently in the process of recruiting patients.

## Abbreviations

ASyMS: Advanced Symptom Management System
CaTS: Cancer Treatment Scale
CTCAE: Common Toxicity Criteria for Adverse Events
CDU: Chemotherapy Day Unit
CLL: chronic lymphocytic leukaemia
CONSORT: Consolidated Standards of Reporting Trials
DM: data manager
ECOG: Eastern Cooperative Oncology Group
EQ-5D: EuroQol-5D
FACT-G: Functional Assessment of Cancer Therapy – General scale
FU: follow up
GP: general practitioner
HADS: Hospital Anxiety and Depression Scale
ICER: incremental cost-effectiveness ratio
MR: medical record
MRC: Medical Research Council
NHL: non-Hodgkin’s lymphoma
PAM: Patient Activation Measure
PRISMS: Patient Remote Intervention and Symptom Management System
QALY: quality-adjusted life year
QOL: health-related quality of life
RCT: randomised controlled trial
RSCL: Rotterdam Symptom Checklist
CTx: chemotherapy treatment
UK: United Kingdom
